# Efficacy and safety of tofacitinib in an open-label, long-term extension study in patients with psoriatic arthritis who received adalimumab or tofacitinib in a Phase 3 randomized controlled study: a post hoc analysis

**DOI:** 10.1186/s13075-024-03442-2

**Published:** 2024-12-19

**Authors:** Dafna D. Gladman, Peter Nash, Philip J. Mease, Oliver FitzGerald, Stephanie Duench, Mary Jane Cadatal, Karim R. Masri

**Affiliations:** 1 Department of Medicine, University of Toronto, and Schroeder Arthritis Institute, Krembil Research Institute, Toronto Western Hospital, 399 Bathurst St. 1E-411, Toronto, ON M5T 2S8 Canada; 2 School of Medicine, Griffith University, Brisbane, QLD Australia; 3 Rheumatology Research, Swedish Medical Center/Providence St. Joseph Health, and University of Washington School of Medicine, Seattle, WA USA; 4 Conway Institute for Biomolecular Research, University College Dublin, Dublin, Ireland; 5 Pfizer Inc, New York City, NY USA; 6 Pfizer Inc, Manila, Philippines

**Keywords:** Psoriatic arthritis, Tofacitinib, Adalimumab, Treatment switching

## Abstract

**Background:**

Data on treatment switching directly from tumor necrosis factor inhibitors to tofacitinib in psoriatic arthritis (PsA) are limited. This post hoc analysis assessed efficacy and safety outcomes in patients with PsA who directly switched to tofacitinib in a long-term extension (LTE) study after receiving adalimumab (ADA) in a Phase 3 study, compared with those who continued to receive tofacitinib.

**Methods:**

Patients with active PsA received tofacitinib 5 mg twice daily (BID) or ADA 40 mg once every 2 weeks in a 12-month, randomized, double-blind study (OPAL Broaden) and then continued or switched to tofacitinib 5 mg BID and maintained this dose in an open-label LTE study (OPAL Balance). Efficacy was assessed 3 months before the last visit and at the last visit in the Phase 3 study, and at month 3 (or month 6 for select outcomes) in the LTE study and included rates of ≥ 20/50/70% improvement in American College of Rheumatology response criteria, Psoriasis Area and Severity Index ≥ 75% improvement, Health Assessment Questionnaire-Disability Index (HAQ-DI) response (decrease from baseline ≥ 0.35 for patients with baseline HAQ-DI ≥ 0.35), Psoriatic Arthritis Disease Activity Score ≤ 3.2, and minimal disease activity; and change from baseline in Functional Assessment of Chronic Illness Therapy-Fatigue score. Safety was assessed at months 3 and 12 in both studies via incidence rates (patients with first events/100 patient-years).

**Results:**

Overall, 180 patients were included (ADA→tofacitinib 5 mg BID: *n* = 91; continuing tofacitinib 5 mg BID: *n* = 89). At Phase 3 baseline, patients in the ADA→tofacitinib 5 mg BID group tended to be younger and have less active disease compared with those continuing tofacitinib. Efficacy was similar between groups in the Phase 3 study, and was maintained to month 3 or 6 in the LTE study. Treatment-emergent adverse events (AEs), serious AEs, and serious infections were generally similar in the Phase 3 and LTE studies, and between groups within each study.

**Conclusion:**

Tofacitinib efficacy and safety were similar in patients with PsA who directly switched from ADA to tofacitinib and those who continued tofacitinib, suggesting that patients can be directly switched from ADA to tofacitinib without any washout period.

**Trial registration:**

NCT01877668; NCT01976364

**Supplementary Information:**

The online version contains supplementary material available at 10.1186/s13075-024-03442-2.

## Background

Treatment of psoriatic arthritis (PsA) typically aims to achieve a target of low disease activity or remission, but a sequence of different treatments may be required to achieve this [1, 2]. Patients with an inadequate response to conventional synthetic disease-modifying antirheumatic drugs (csDMARDs) are recommended to be treated with biological DMARDs (bDMARDs) such as tumor necrosis factor inhibitors (TNFi) [1, 2]. However, the efficacy of bDMARDs may also be lost due to the formation of antidrug antibodies [3, 4]. In a retrospective claims analysis of US patients with PsA, 89% initiated a TNFi as the first advanced treatment but approximately one-third of patients switched to another advanced treatment within two years [5]. Similarly, in a UK registry study, only 61% of patients initiating a TNFi remained on the same drug after three years [6].

Patients who do not respond to a bDMARD or experience adverse events (AEs) may switch to another drug within the same class or one with an alternative mechanism of action, such as a Janus kinase (JAK) inhibitor. The European Alliance of Associations for Rheumatology (EULAR) recommendations for pharmacological treatment of PsA suggest JAK inhibitors for patients with peripheral arthritis and inadequate response to ≥ 1 bDMARD, with caution recommended for patients with safety risk factors [1]. The Group for Research and Assessment of Psoriasis and Psoriatic Arthritis (GRAPPA) also recommended JAK inhibitors as a treatment of PsA for patients with peripheral arthritis who were csDMARD-naïve or had inadequate response to ≥ 1 csDMARD or bDMARD, or patients with enthesitis, dactylitis, or psoriasis [2].

Tofacitinib is an oral JAK inhibitor for the treatment of PsA. The efficacy and safety of tofacitinib in PsA has been demonstrated in two Phase 3 studies [7, 8] and a long-term extension (LTE) study [9]. However, there are no available data from randomized controlled trials in PsA assessing clinical outcomes in patients who have directly switched from TNFi to tofacitinib, and whether they maintain or achieve disease control with an acceptable safety profile. Adalimumab (ADA), a TNFi, was included as an active control in the tofacitinib Phase 3 OPAL Broaden study [7]. Patients could receive ADA in OPAL Broaden and subsequently switch to tofacitinib in the LTE study [9]. Given the different mechanisms of action of these treatments [10, 11], and the long half-life of ADA (15–19 days) [12], it is possible that directly switching from ADA to tofacitinib with no washout period could result in a short period of overlapping immunomodulatory effects. However, to maintain disease control, it may be preferable to avoid a washout period as discontinuing DMARD treatment can lead to a rapid increase in PsA symptoms [13]. Therefore, the objective of this post hoc analysis was to assess efficacy and safety outcomes in patients with PsA who directly switched to tofacitinib after receiving ADA, compared with those who continued to receive tofacitinib.

## Methods

### Study design and patients

This post hoc analysis included data from a 12-month, randomized, double-blind Phase 3 study (NCT01877668; OPAL Broaden) [7] and a 36-month, open-label LTE study (NCT01976364; OPAL Balance) [9]. Details of the study design and eligibility criteria have been described previously.

Briefly, the Phase 3 study enrolled patients aged ≥ 18 years with a diagnosis of PsA for at least 6 months and active arthritis and plaque psoriasis [7]. Patients had an inadequate response to ≥ 1 csDMARD and had not previously received a TNFi. Eligible patients were randomized to receive tofacitinib 5 mg twice daily (BID), tofacitinib 10 mg BID, ADA 40 mg once every 2 weeks, placebo with a switch to tofacitinib 5 mg BID at month 3, or placebo with a switch to tofacitinib 10 mg BID at month 3, all taken with a csDMARD.

Patients who completed the Phase 3 study or discontinued for reasons other than an AE related to treatment were eligible to participate in the LTE study, in which all patients received tofacitinib 5 mg BID. The dose could be increased to 10 mg BID after 1 month for inadequate symptom control, and thereafter reduced to 5 mg BID for safety reasons. Patients who entered the LTE study from the ADA group of the Phase 3 study received their first dose of tofacitinib ≥ 1 week after their last ADA injection. The LTE study also enrolled patients from OPAL Beyond (NCT01882439), but those patients were not included in this analysis.

Both studies were conducted in accordance with the Good Clinical Practice guidelines of the International Council for Harmonisation and with the principles of the Declaration of Helsinki. All patients provided written informed consent and the protocols were approved by the institutional review board or independent ethics committee at each investigational site.

### Post hoc analysis outcomes

#### Efficacy

The proportions of patients achieving ≥ 20%, ≥ 50%, and ≥ 70% improvements in American College of Rheumatology response criteria (ACR20, ACR50, and ACR70 responses, respectively), ≥ 75% Psoriasis Area and Severity Index improvement from baseline (PASI75) response, Health Assessment Questionnaire-Disability Index (HAQ-DI) response (decrease from baseline ≥ 0.35 for patients with baseline HAQ-DI ≥ 0.35), Psoriatic Arthritis Disease Activity Score (PASDAS) ≤ 3.2, minimal disease activity (MDA) response, and very low disease activity (VLDA) response were reported. Mean changes from the Phase 3 study baseline in Functional Assessment of Chronic Illness Therapy-Fatigue (FACIT-F) total scores, HAQ-DI scores, Physician’s Global Assessment of Psoriasis (PGA-PsO) scores, Patient Assessment of Arthritis Pain scores (on a visual analog scale [VAS]), and PASDAS were also assessed.

Efficacy outcomes were assessed at 3 months before the last visit and at the last visit in the Phase 3 study, and at month 3 (or month 6 for PASDAS and FACIT-F outcomes) in the LTE study.

### Safety

Safety outcomes of interest included proportions of patients with events and incidence rates (IRs; patients with first events/100 patient-years [PY]) for treatment-emergent AEs (TEAEs), serious AEs (SAEs), serious infections, infections (assessed as a System Organ Class [SOC] from the Medical Dictionary for Regulatory Activities [MedDRA]), herpes zoster, opportunistic infections, tuberculosis, malignancies excluding non-melanoma skin cancer (NMSC), NMSC, lymphoma, major adverse cardiovascular events (MACE), venous thromboembolism, and all-cause deaths. The most frequently occurring TEAEs (occurring in ≥ 5% of patients in any group) were tabulated by MedDRA Preferred Term. AEs were coded using MedDRA v22.0. IRs and proportions for safety outcomes were assessed from months 0–3 and 0–12 in both studies.

Changes from baseline in aspartate aminotransferase (AST), alanine aminotransferase (ALT), low-density lipoprotein cholesterol, high-density lipoprotein cholesterol, total cholesterol, triglycerides, lymphocytes, neutrophils, and hemoglobin were assessed at months 3, 6, and 12 in the Phase 3 and LTE studies. Proportions of patients exceeding upper limit of normal (ULN) thresholds in total bilirubin, AST, ALT, and gamma glutamyl transferase (GT) were tabulated.

### Statistical analysis

For this post hoc analysis, patients were analyzed in two groups. The ADA→tofacitinib 5 mg BID group included patients who received ADA in the Phase 3 study and then directly switched to tofacitinib 5 mg BID and maintained this dose in the LTE study. The continuing tofacitinib 5 mg BID group included patients who received tofacitinib 5 mg BID in the Phase 3 study and then continued tofacitinib 5 mg BID and maintained this dose in the LTE study.

The LTE safety analysis set included patients from the Phase 3 study who had at least one dose of treatment in the LTE study. Patients with a > 14-day gap in treatment between the last observation in the Phase 3 study and the first dose of tofacitinib in the LTE study were excluded from any ‘by-visit’ summaries of continuous data (i.e., laboratory variables), and evaluation of all efficacy endpoints also followed this 14-day gap rule.

Demographic and disease characteristics at Phase 3 study baseline were summarized descriptively. For efficacy outcomes, proportions were calculated based on the number of patients with non-missing responses at each visit. Least squares mean changes from baseline were analyzed using a repeated measures model with the fixed effects of treatment, visit, treatment by visit interaction, geographic location, and baseline value. An unstructured covariance matrix was used. No formal statistical testing was conducted for this post hoc analysis; consequently, *p*-values for the comparisons between treatment groups are nominal and are provided for descriptive purposes only. As an additional descriptive analysis, for dichotomous response outcomes, patients in the ADA→tofacitinib 5 mg BID group were categorized based on their response to ADA at the last Phase 3 visit and their subsequent response to tofacitinib at month 3 in the LTE study.

For IRs of safety outcomes, follow-up time was calculated up to the day of the first event, subject to a risk period of 28 days beyond the last dose or to the data cut-off date. Gaps in dosing between treatment switches or between the Phase 3 and LTE studies were included up to 28 days or to the data cut-off date. Exact Poisson (adjusted for PY) 95% confidence intervals (CIs) were calculated for the crude IRs. Laboratory parameters were reported using descriptive statistics.

Data were reported as observed, with no imputation for missing data.

## Results

### Patients

In total, 180 patients who participated in the Phase 3 and LTE studies were included in this analysis. Of these, 91 (50.6%) patients switched from ADA to tofacitinib 5 mg BID and 89 (49.4%) patients continued on tofacitinib 5 mg BID in the LTE study.

Baseline characteristics across groups are presented in Table 1. A higher percentage of patients in the continuing tofacitinib 5 mg BID group were ≥ 65 years of age compared with the ADA→tofacitinib 5 mg BID group. Also, patients in the continuing tofacitinib 5 mg BID group had higher mean swollen/tender joint counts and Patient Assessment of Arthritis Pain scores at baseline than the ADA→tofacitinib 5 mg BID group. Furthermore, the proportions of patients with concomitant use of methotrexate and prior use of corticosteroids were higher in the continuing tofacitinib 5 mg BID group than in the ADA→tofacitinib 5 mg BID group.

**Table 1:** Patient demographics and disease characteristics at Phase 3 study baseline

	ADA→ tofacitinib 5 mg BID (*N* = 91)	Continuing tofacitinib 5 mg BID (*N* = 89)	Total (*N* = 180)
Age in years, median (range)	46.0 (27.0–70.0)	51.0 (23.0–72.0)	48.0 (23.0–72.0)
Age ≥ 65 years, *n* (%)	3 (3.3)	10 (11.2)	13 (7.2)
Female, *n* (%)	44 (48.4)	47 (52.8)	91 (50.6)
White, *n* (%)	88 (96.7)	88 (98.9)	176 (97.8)
BMI (kg/m^2^), mean (SD)	28.6 (5.1)	29.2 (5.5)	28.9 (5.3)
Disease duration (years), mean (SD)	5.3 (5.0)	6.6 (7.2)	5.9 (6.2)
Swollen joint count (of 66 joints), mean (SD)	9.2 (5.8)	13.3 (10.3)	11.2 (8.6)
Tender joint count (of 68 joints), mean (SD)	16.4 (10.7)	20.6 (12.8)	18.5 (12.0)
LEI score > 0, *n* (%)	64 (70.3)	61 (68.5)	125 (69.4)
LEI score, mean (SD)	2.2 (1.2)	2.5 (1.3)	2.3 (1.3)
DSS > 0, *n* (%)	50 (54.9)	50 (56.2)	100 (55.6)
DSS, mean (SD)	7.0 (5.8)	8.6 (8.2)	7.8 (7.1)
HAQ-DI, mean (SD)	1.0 (0.6)	1.2 (0.6)	1.1 (0.6)
PASI in patients with BSA ≥ 3% and PASI > 0, mean (SD)	10.2 (8.9)	8.1 (7.9)	9.1 (8.5)
PASDAS, mean (SD)	5.8 (1.2)	6.0 (1.1)	5.9 (1.2)
PGA-PsO in patients with PGA-PsO > 0, mean (SD)	1.9 (0.7)	2.0 (0.8)	1.9 (0.8)
Patient Assessment of Arthritis Pain (VAS), mean (SD)	49.3 (21.7)	56.0 (23.1)	52.6 (22.6)
FACIT-F, mean (SD)	30.7 (11.1)	28.0 (10.2)	29.4 (10.7)
Concomitant methotrexate, *n* (%)	69 (75.8)	77 (86.5)	146 (81.1)
Oral corticosteroid use, *n* (%)	21 (23.1)	26 (29.2)	47 (26.1)

ADA: adalimumab, BID: twice daily, BMI: body mass index, BSA: body surface area, DSS: dactylitis severity score, FACIT-F: Functional Assessment of Chronic Illness Therapy-Fatigue, HAQ-DI: Health Assessment Questionnaire-Disability Index, LEI: Leeds Enthesitis Index, *N*: number of evaluable patients; *n*: number of patients with characteristic, PASDAS: Psoriatic Arthritis Disease Activity Score, PASI: Psoriasis Area and Severity Index, PGA-PsO: Physician’s Global Assessment of Psoriasis, SD: standard deviation, VAS: visual analog scale

### Efficacy in the Phase 3 and LTE studies

**Fig. 1:**
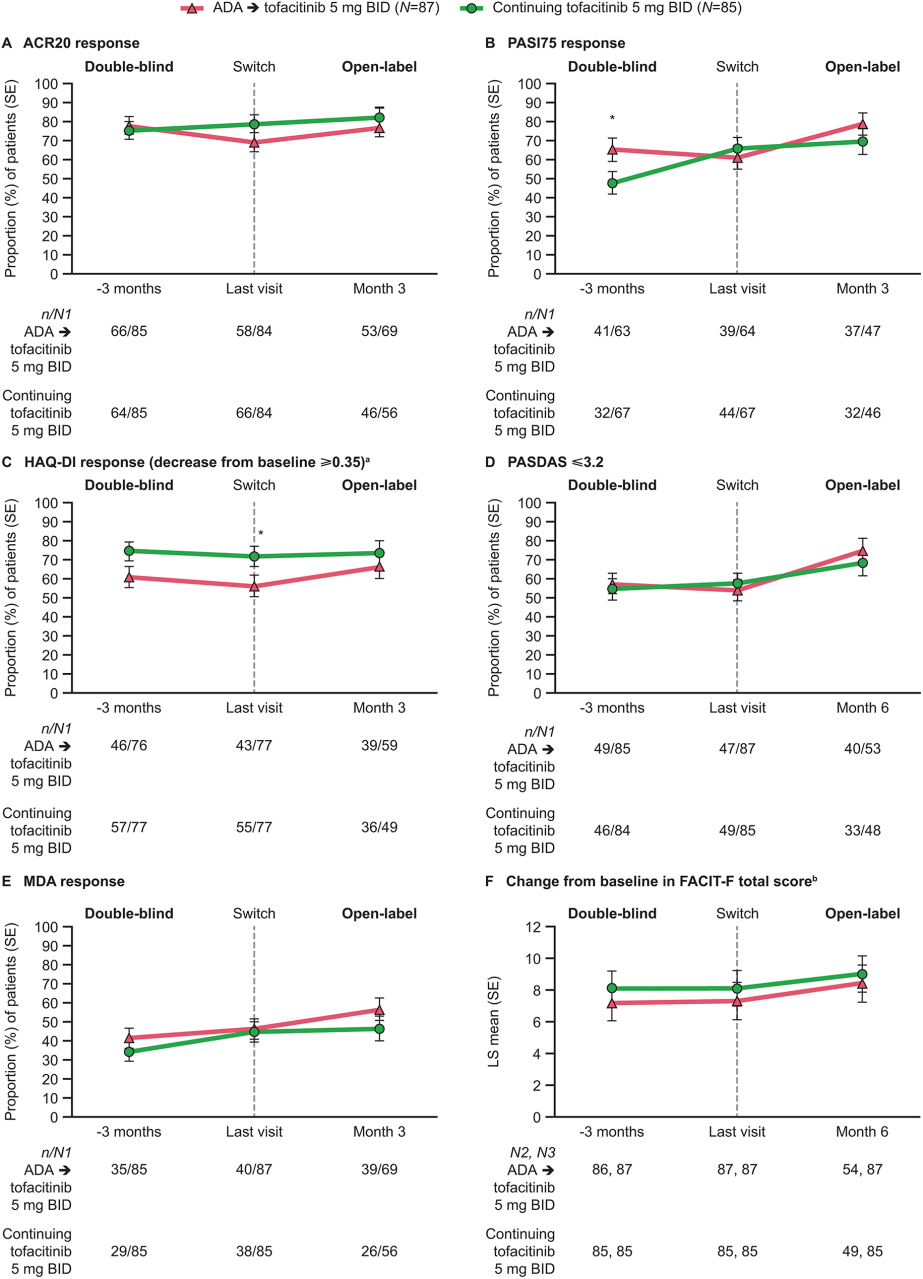
Efficacy outcomes in the Phase 3 and LTE studies. *p < 0.05 for comparison between treatment groups. -3 months: 3 months prior to the last visit in the Phase 3 study. Last visit: the last visit in the Phase 3 study. Month 3: month 3 in the LTE study. Month 6: month 6 in the LTE study. Baseline refers to the baseline visit of the Phase 3 study. ^a^In patients with baseline HAQ-DI ≥ 0.35 (*N* = 77). ^b^Results are based on a repeated measures model with the fixed effects of treatment, visit, treatment by visit interaction, geographic location, and baseline value – an unstructured covariance matrix was used. ACR20: ≥ 20% improvement in American College of Rheumatology response criteria, ADA: adalimumab, BID: twice daily, FACIT-F: Functional Assessment of Chronic Illness Therapy-Fatigue, HAQ-DI: Health Assessment Questionnaire-Disability Index, LS: least squares, LTE: long-term extension, MDA: minimal disease activity, *N*: number of patients in the LTE safety analysis set, *n*: number of patients with response, *N1*: number of patients with non-missing response at visit, *N2*: number of patients with observations at visit, *N3*: number of patients included in the mixed model for repeated measures, *PASDAS*: Psoriatic Arthritis Disease Activity Score, PASI75: ≥ 75% Psoriasis Area and Severity Index improvement from baseline, SE: standard error

The proportions of patients achieving ACR20 and ACR50 responses were similar between the ADA→tofacitinib 5 mg BID and continuing tofacitinib 5 mg BID groups during the last 3 months of the Phase 3 study (Fig. 1A, Supplementary Fig. 1A). Responses were maintained to month 3 in the LTE study and remained similar in both groups. Similarly, ACR70 responses were maintained from the Phase 3 study to the LTE study in both groups, although response rates were numerically higher in the ADA→tofacitinib 5 mg BID group than in the continuing tofacitinib 5 mg BID group (Supplementary Fig. 1B).

A greater proportion of patients in the ADA→tofacitinib 5 mg BID group, compared with the continuing tofacitinib 5 mg BID group, had achieved a PASI75 response 3 months before the last Phase 3 visit (*p* < 0.05), but similar proportions of patients in both groups achieved response at the last Phase 3 visit and at month 3 in the LTE study (Fig. 1B). There were no differences between treatment groups for the mean change from baseline in PGA-PsO scores, and both groups continued to show numerical improvements from baseline in the first 3 months of the LTE study (Supplementary Fig. 1C).

In the last 3 months of the Phase 3 study, HAQ-DI response rates and mean changes from baseline in HAQ-DI were numerically larger in the continuing tofacitinib 5 mg BID group than in the ADA→tofacitinib 5 mg BID group (*p* < 0.05 for response rate at the last Phase 3 visit), but by month 3 in the LTE study, results were similar for both groups (Fig. 1C, Supplementary Fig. 1D).

The proportion of patients with a PASDAS ≤ 3.2 and changes from baseline in PASDAS were similar between treatment groups from the last 3 months of the Phase 3 study through to month 6 in the LTE study, and increased during the LTE study in both groups (Fig. 1D, Supplementary Fig. 1E).

Mean changes from baseline in Patient Assessment of Arthritis Pain (VAS) were similar between treatment groups from the last 3 months of the Phase 3 study through to the month 3 of the LTE study (Supplementary Fig. 1F).

MDA response rates were similar between treatment groups at all three time points assessed (Fig. 1E). VLDA response rates were higher in the ADA→tofacitinib 5 mg BID group than in the continuing tofacitinib 5 mg BID group at the last visit in the Phase 3 study (*p* < 0.05), but were similar between groups by month 3 of the LTE study (Fig. 1E, Supplementary Fig. 1G).

Mean changes from baseline in FACIT-F total scores were generally similar between groups, and scores improved during the first 6 months of the LTE study in both groups (Fig. 1F).

**Fig. 2:**
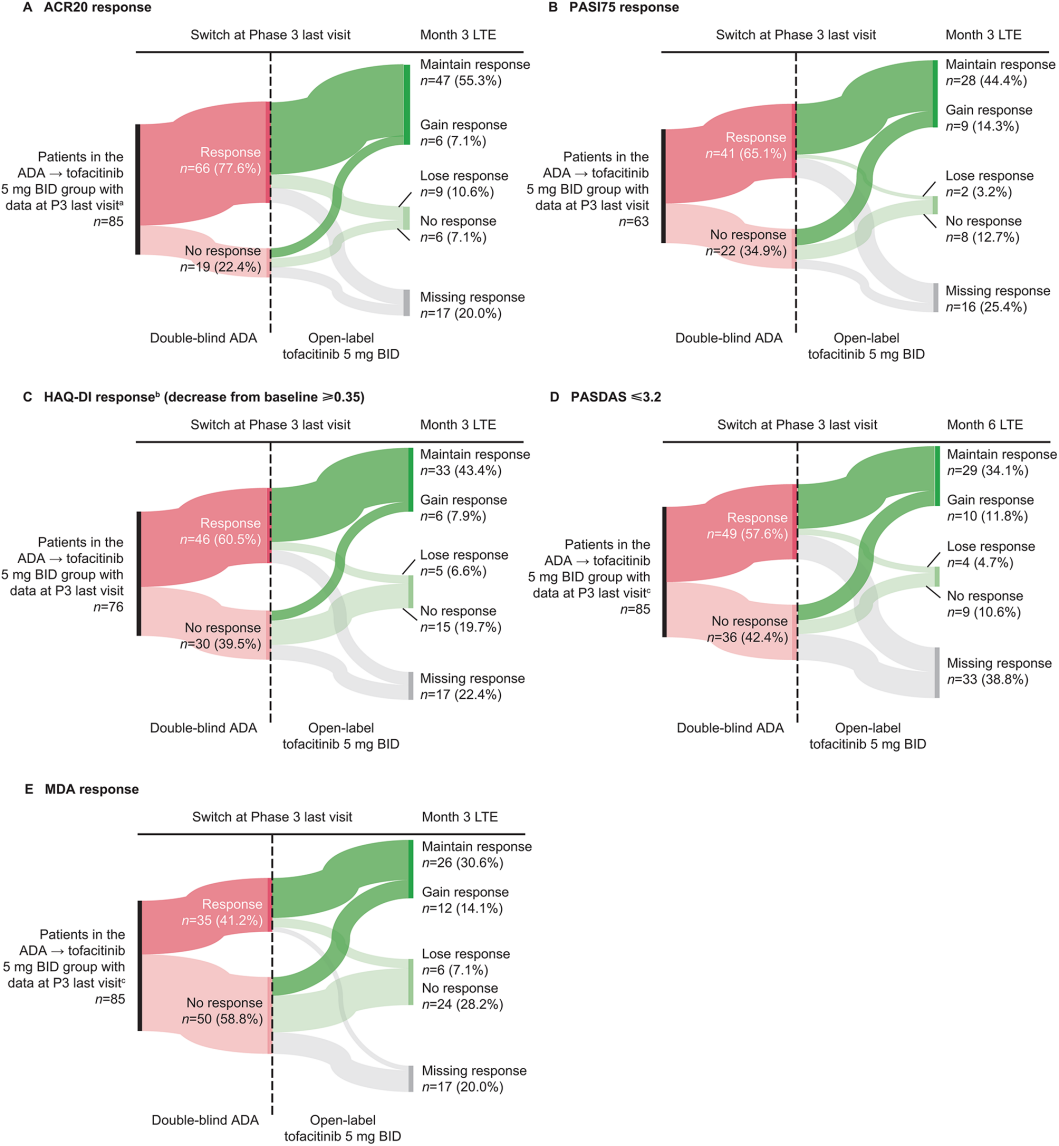
Efficacy outcomes after switching from ADA→tofacitinib 5 mg BID according to Phase 3 ADA response. Efficacy outcomes for patients in the ADA→tofacitinib 5 mg BID group are shown following switch to tofacitinib in the LTE study according to ADA response in the Phase 3 study. ^a^Figure excludes one patient in the ADA→tofacitinib 5 mg BID group who had a missing response to ADA at the Phase 3 last visit and no response to tofacitinib at the month 3 LTE. ^b^In patients with baseline HAQ-DI ≥ 0.35. ^c^Figure excludes one patient in the ADA→tofacitinib 5 mg BID group who had a missing response to ADA at the Phase 3 last visit and a response to tofacitinib at the month 3 LTE. ACR20: ≥ 20% improvement in American College of Rheumatology response criteria, ADA: adalimumab, BID: twice daily, HAQ-DI: Health Assessment Questionnaire-Disability Index, LTE: long-term extension, MDA: minimal disease activity, *n*: number of patients in category, P3: Phase 3, PASDAS: Psoriatic Arthritis Disease Activity Score, PASI75: ≥ 75% Psoriasis Area and Severity Index improvement from baseline

In the descriptive analyses of treatment responses in the ADA→tofacitinib 5 mg BID group, most patients who achieved ACR20/50/70 response, PASI75 response, HAQ-DI response, PASDAS ≤ 3.2, and MDA or VLDA response while receiving ADA in the Phase 3 study also achieved responses for these outcomes at month 3 in the LTE study with tofacitinib (Fig. 2 and Supplementary Fig. 2). Smaller proportions of patients only achieved responses with tofacitinib in the LTE study after switching (no response with ADA at the last Phase 3 visit) or only achieved response with ADA in the Phase 3 study (Fig. 2 and Supplementary Fig. 2).

### Safety in the Phase 3 and LTE studies

**Fig. 3:**
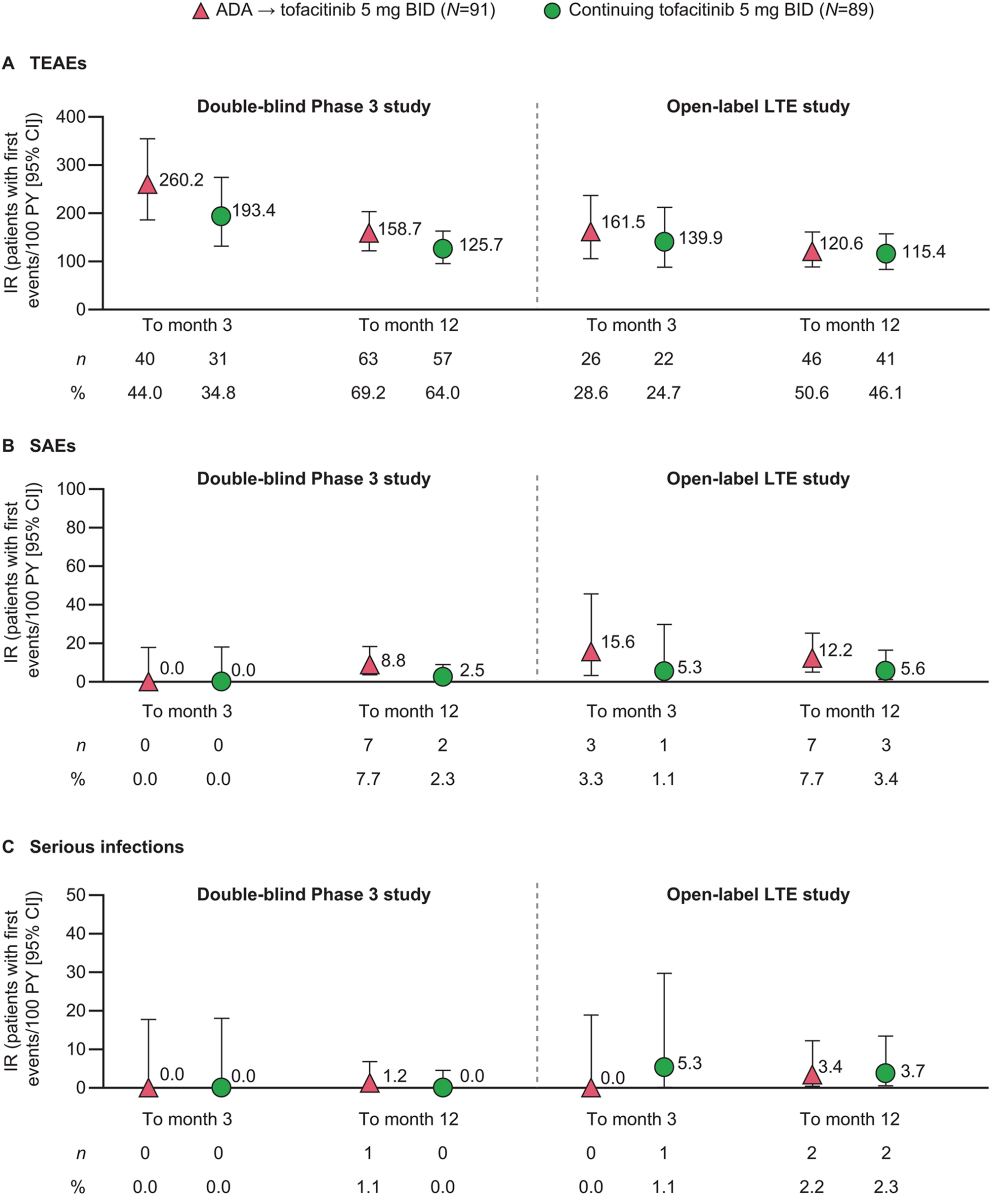
Incidence of safety outcomes in the Phase 3 and LTE studies. Total follow-up time calculated up to the first day of the first event, subject to a risk period of 28 days beyond the last dose or to the data cut-off date. Gaps in dosing between treatment switches or between the Phase 3 and LTE studies are included up to 28 days or to the data cut-off date. ADA: adalimumab, BID: twice daily, CI: confidence interval, IR: incidence rate, LTE: long-term extension, *N*: number of evaluable patients, *n*: number of patients with events, SAE: serious adverse event, TEAE: treatment-emergent adverse event

The incidence of TEAEs was generally similar between treatment groups throughout both studies; while IRs were numerically higher in the ADA→tofacitinib 5 mg BID group compared with the continuing tofacitinib 5 mg BID group before and after the treatment switch, 95% CIs overlapped (Fig. 3A). In both treatment groups, the IRs for TEAEs were higher in the first 3 months of each study, compared with the full 12 months, and no increase in TEAEs was observed after switching in the ADA→ tofacitinib 5 mg BID group. IRs of SAEs and serious infections were slightly higher in the LTE study than in the Phase 3 study in both treatment groups, but the number of events overall was low (Fig. 3B–C).

The IR for infections was numerically higher in the continuing tofacitinib 5 mg BID group than in the ADA→tofacitinib 5 mg BID group over the first 3 months of the Phase 3 study, though with overlapping 95% CIs, but similar between groups over 12 months in the Phase 3 study and in the LTE study (Table 2). A low number of herpes zoster, opportunistic infections, malignancies excluding NMSC, NMSC, and MACE events occurred, which were too small for further interpretation by treatment group (Table 2). There were no events of tuberculosis, lymphoma, venous thromboembolism, or death in either treatment group.

**Table 2:** Incidence of additional safety outcomes in the Phase 3 and LTE studies

	Phase 3 study				LTE study			
**Interval**	**To month 3**		**To month 12**		**To month 3**		**To month 12**	
	**ADA**→ **tofacitinib ** **5 mg BID (*N*** **= 91)**	**Continuing tofacitinib ** **5 mg BID (*N*** **= 89)**	**ADA**→ **tofacitinib ** **5 mg BID (*N*** **= 91)**	**Continuing tofacitinib ** **5 mg BID (*N*** **= 89)**	**ADA**→ **tofacitinib ** **5 mg BID (*N*** **= 91)**	**Continuing tofacitinib ** **5 mg BID (*N*** **= 89)**	**ADA**→ **tofacitinib ** **5 mg BID (*N*** **= 91)**	**Continuing tofacitinib ** **5 mg BID (*N*** **= 89)**
**Infections**								
*n* (%)	10 (11.0)	14 (15.7)	36 (39.6)	34 (38.2)	8 (8.8)	7 (7.9)	22 (24.2)	21 (23.6)
IR (95% CI)	50.3 (24.1–92.5)	74.6 (40.8–125.1)	55.3 (38.7–76.5)	53.9 (37.3–75.3)	43.0 (18.6–84.7)	39.2 (15.8–80.8)	43.2 (27.1–65.4)	46.8 (29.0–71.5)
**Herpes zoster**								
*n* (%)	0 (0.0)	1 (1.1)	0 (0.0)	2 (2.3)	1 (1.1)	1 (1.1)	1 (1.1)	1 (1.1)
IR (95% CI)	0.0 (0.0–17.7)	4.9 (0.1–27.3)	0.0 (0.0–4.5)	2.5 (0.3–9.0)	5.2 (0.1–28.7)	5.3 (0.1–29.7)	1.7 (0.0–9.5)	1.9 (0.1–10.3)
**Opportunistic infection**								
*n* (%)	0 (0.0)	0 (0.0)	0 (0.0)	0 (0.0)	1 (1.1)	0 (0.0)	1 (1.1)	0 (0.0)
IR (95% CI)	0.0 (0.0–17.7)	0.0 (0.0–18.0)	0.0 (0.0–4.5)	0.0 (0.0–4.5)	5.2 (0.1–28.7)	0.0 (0.0–19.7)	1.7 (0.0–9.5)	0.0 (0.0–6.8)
**Malignancies excluding NMSC**								
*n* (%)	0 (0.0)	0 (0.0)	0 (0.0)	0 (0.0)	1 (1.1)	0 (0.0)	2 (2.2)	0 (0.0)
IR (95% CI)	0.0 (0.0–17.7)	0.0 (0.0–18.0)	0.0 (0.0–4.5)	0.0 (0.0–4.5)	5.1 (0.1–28.7)	0.0 (0.0–19.7)	3.4 (0.4–12.2)	0.0 (0.0–6.8)
**NMSC**								
*n* (%)	0 (0.0)	0 (0.0)	0 (0.0)	0 (0.0)	0 (0.0)	1 (1.1)	0 (0.0)	1 (1.1)
IR (95% CI)	0.0 (0.0–17.7)	0.0 (0.0–18.0)	0.0 (0.0–4.5)	0.0 (0.0–4.5)	0.0 (0.0–18.9)	5.4 (0.1–29.9)	0.0 (0.0–6.2)	1.9 (0.1–10.5)
**MACE**								
*n* (%)	0 (0.0)	0 (0.0)	0 (0.0)	0 (0.0)	0 (0.0)	0 (0.0)	1 (1.1)	0 (0.0)
IR (95% CI)	0.0 (0.0–17.7)	0.0 (0.0–18.0)	0.0 (0.0–4.5)	0.0 (0.0–4.5)	0.0 (0.0–18.9)	0.0 (0.0–19.7)	1.7 (0.0–9.4)	0.0 (0.0–6.8)
**Tuberculosis**								
*n* (%)	0 (0.0)	0 (0.0)	0 (0.0)	0 (0.0)	0 (0.0)	0 (0.0)	0 (0.0)	0 (0.0)
IR (95% CI)	0.0 (0.0–17.7)	0.0 (0.0–18.0)	0.0 (0.0–4.5)	0.0 (0.0–4.5)	0.0 (0.0–18.9)	0.0 (0.0–19.7)	0.0 (0.0–6.2)	0.0 (0.0–6.8)
**Lymphoma**								
*n* (%)	0 (0.0)	0 (0.0)	0 (0.0)	0 (0.0)	0 (0.0)	0 (0.0)	0 (0.0)	0 (0.0)
IR (95% CI)	0.0 (0.0–17.7)	0.0 (0.0–18.0)	0.0 (0.0–4.5)	0.0 (0.0–4.5)	0.0 (0.0–18.9)	0.0 (0.0–19.7)	0.0 (0.0–6.2)	0.0 (0.0–6.8)
**Venous thromboembolism**								
*n* (%)	0 (0.0)	0 (0.0)	0 (0.0)	0 (0.0)	0 (0.0)	0 (0.0)	0 (0.0)	0 (0.0)
IR (95% CI)	0.0 (0.0–17.7)	0.0 (0.0–18.0)	0.0 (0.0–4.5)	0.0 (0.0–4.5)	0.0 (0.0–18.9)	0.0 (0.0–19.7)	0.0 (0.0–6.2)	0.0 (0.0–6.8)
**All-cause death**								
*n* (%)	0 (0.0)	0 (0.0)	0 (0.0)	0 (0.0)	0 (0.0)	0 (0.0)	0 (0.0)	0 (0.0)
IR (95% CI)	0.0 (0.0–17.7)	0.0 (0.0–18.0)	0.0 (0.0–4.5)	0.0 (0.0–4.5)	0.0 (0.0–18.9)	0.0 (0.0–19.7)	0.0 (0.0–6.2)	0.0 (0.0–6.8)

Total follow-up time calculated up to the first day of the first event, subject to a risk period of 28 days beyond the last dose or to the data cut-off date. Gaps in dosing between treatment switches or between the Phase 3 and LTE studies are included up to 28 days or to the data cut-off date

ADA: adalimumab, BID: twice daily, CI: confidence interval, IR: incidence rate, LTE: long-term extension, MACE: major adverse cardiovascular event, *N*: number of evaluable patients, *n*: number of patients with events, NMSC: non-melanoma skin cancer, SOC, System Organ Class

The most common TEAEs across both studies were nasopharyngitis and upper respiratory tract infection (Table 3).

**Table 3:** TEAEs by Preferred Term^a^ occurring in ≥ 5% of patients in any group in the Phase 3 and LTE studies

	Phase 3 study				LTE study			
**Interval**	**To month 3**		**To month 12**		**To month 3**		**To month 12**	
	**ADA**→ **tofacitinib ** **5 mg BID (*N*** **= 91)**	**Continuing tofacitinib ** **5 mg BID (*N*** **= 89)**	**ADA**→ **tofacitinib ** **5 mg BID (*N*** **= 91)**	**Continuing tofacitinib ** **5 mg BID (*N*** **= 89)**	**ADA**→ **tofacitinib ** **5 mg BID (*N*** **= 91)**	**Continuing tofacitinib ** **5 mg BID (*N*** **= 89)**	**ADA**→ **tofacitinib ** **5 mg BID (*N*** **= 91)**	**Continuing tofacitinib ** **5 mg BID (*N*** **= 89)**
**ALT increased**								
*n* (%)	4 (4.4)	1 (1.1)	7 (7.7)	3 (3.4)	1 (1.1)	0 (0.0)	1 (1.1)	0 (0.0)
IR (95% CI)	19.7 (5.4–50.3)	4.9 (0.1–27.4)	9.1 (3.6–18.7)	3.7 (0.8–10.9)	5.2 (0.1–28.8)	0.0 (0.0–19.7)	1.7 (0.0–9.4)	0.0 (0.0–6.8)
**AST increased**								
*n* (%)	3 (3.3)	0 (0.0)	7 (7.7)	0 (0.0)	1 (1.1)	0 (0.0)	1 (1.1)	0 (0.0)
IR (95% CI)	14.7 (3.0–43.1)	0.0 (0.0–18.0)	9.0 (3.6–18.6)	0.0 (0.0–4.5)	5.2 (0.1–28.8)	0.0 (0.0–19.7)	1.7 (0.0–9.4)	0.0 (0.0–6.8)
**Blood creatine phosphokinase increased**								
*n* (%)	2 (2.2)	1 (1.1)	3 (3.3)	5 (5.6)	1 (1.1)	1 (1.1)	1 (1.1)	3 (3.4)
IR (95% CI)	9.8 (1.2–35.2)	4.9 (0.1–27.3)	3.8 (0.8–11.0)	6.3 (2.0–14.7)	5.2 (0.1–28.8)	5.4 (0.1–30.1)	1.7 (0.0–9.4)	5.7 (1.2–16.8)
**Headache**								
*n* (%)	5 (5.5)	3 (3.4)	7 (7.7)	4 (4.5)	0 (0.0)	1 (1.1)	0 (0.0)	2 (2.3)
IR (95% CI)	24.9 (8.1–58.0)	15.0 (3.1–43.9)	9.1 (3.7–18.7)	5.1 (1.4–13.0)	0.0 (0.0–18.9)	5.4 (0.1–30.0)	0.0 (0.0–6.2)	3.8 (0.5–13.6)
**Hypertension**								
*n* (%)	1 (1.1)	0 (0.0)	4 (4.4)	1 (1.1)	2 (2.2)	0 (0.0)	6 (6.6)	2 (2.3)
IR (95% CI)	4.9 (0.1–27.0)	0.0 (0.0–18.0)	5.0 (1.4–12.9)	1.2 (0.0–6.9)	10.5 (1.3–37.8)	0.0 (0.0–19.7)	10.7 (3.9–23.3)	3.8 (0.5–13.6)
**Injection-site erythema**								
*n* (%)	5 (5.5)	0 (0.0)	5 (5.5)	0 (0.0)	0 (0.0)	0 (0.0)	0 (0.0)	0 (0.0)
IR (95% CI)	24.8 (8.0–57.8)	0.0 (0.0–18.0)	6.4 (2.1–15.0)	0.0 (0.0–4.5)	0.0 (0.0–18.9)	0.0 (0.0–19.7)	0.0 (0.0–6.2)	0.0 (0.0–6.8)
**Nasopharyngitis**								
*n* (%)	5 (5.5)	3 (3.4)	9 (9.9)	6 (6.7)	2 (2.2)	0 (0.0)	8 (8.8)	2 (2.3)
IR (95% CI)	24.6 (8.0–57.4)	14.9 (3.1–43.6)	11.8 (5.4–22.4)	7.6 (2.8–16.6)	10.4 (1.3–37.5)	0.0 (0.0–19.7)	14.1 (6.1–27.8)	3.8 (0.5–13.6)
**Pharyngitis**								
*n* (%)	1 (1.1)	0 (0.0)	7 (7.7)	4 (4.5)	1 (1.1)	0 (0.0)	2 (2.2)	0 (0.0)
IR (95% CI)	4.8 (0.1–26.8)	0.0 (0.0–18.0)	8.8 (3.6–18.2)	5.0 (1.4–12.7)	5.2 (0.1–28.9)	0.0 (0.0–19.7)	3.4 (0.4–12.4)	0.0 (0.0–6.8)
**Psoriasis**								
*n* (%)	0 (0.0)	0 (0.0)	3 (3.3)	3 (3.4)	1 (1.1)	0 (0.0)	7 (7.7)	1 (1.1)
IR (95% CI)	0.0 (0.0–17.7)	0.0 (0.0–18.0)	3.7 (0.8–10.9)	3.7 (0.8–10.9)	5.1 (0.1–28.6)	0.0 (0.0–19.7)	12.2 (4.9–25.1)	1.9 (0.1–10.4)
**Upper respiratory tract infection**								
*n* (%)	2 (2.2)	2 (2.3)	7 (7.7)	10 (11.2)	2 (2.2)	1 (1.1)	4 (4.4)	5 (5.6)
IR (95% CI)	9.8 (1.2–35.2)	9.9 (1.2–35.8)	8.9 (3.6–18.3)	13.0 (6.2–23.9)	10.4 (1.3–37.4)	5.4 (0.1–29.9)	6.9 (1.9–17.5)	9.7 (3.2–22.7)

^a^MedDRA (v22.0) coding dictionary applied

Total follow-up time calculated up to the first day of the first event, subject to a risk period of 28 days beyond the last dose or to the data cut-off date. Gaps in dosing between treatment switches or between the Phase 3 and LTE studies are included up to 28 days or to the data cut-off date

ADA: adalimumab, ALT: alanine aminotransferase, AST: aspartate aminotransferase, BID: twice daily, CI: confidence interval, IR: incidence rate, LTE: long-term extension, MedDRA: Medical Dictionary for Regulatory Activities, *N*: number of evaluable patients, *n*: number of patients with events, TEAE: treatment-emergent adverse event

Changes from baseline in laboratory parameters throughout the Phase 3 and LTE studies are shown in Supplementary Fig. 3. Patients who received ADA in the Phase 3 study (i.e., those in the ADA→tofacitinib 5 mg BID group) had larger increases in lymphocytes and hemoglobin, and larger decreases in neutrophils, than patients in the continuing tofacitinib 5 mg BID group (Supplementary Fig. 3G–I). By month 12 in the LTE study, when both groups were receiving tofacitinib, these treatment differences were reduced.

Elevations in total bilirubin, AST, ALT, and gamma GT above the ULN (using thresholds of > 1, ≥ 2, ≥ 3, ≥ 5, or ≥ 10x) were more frequent in patients receiving ADA than tofacitinib 5 mg BID during the Phase 3 study, but were generally similar between groups during the LTE study following the treatment switch (Supplementary Table 1).

## Discussion

In this post hoc analysis of a Phase 3 study followed by an LTE study (OPAL Broaden and OPAL Balance, respectively), the impact of directly switching from ADA to tofacitinib was assessed in patients with PsA. Patients who continued to receive tofacitinib throughout both trials were included as a comparison group, allowing for assessment of any effects of transitioning from the double-blind Phase 3 study to the open-label LTE study. Overall, efficacy and safety outcomes were generally similar between the two groups, suggesting that patients with PsA can be directly switched from ADA to tofacitinib without any washout period.

While no data are available assessing the impact of direct switching between these treatments in PsA, a study in patients with rheumatoid arthritis (RA) explored the safety and efficacy of open-label tofacitinib following blinded treatment with ADA or tofacitinib, similar to the design of the current analysis [14]. In the RA study, a similar safety and efficacy profile was seen when patients received open-label tofacitinib after receiving either blinded ADA or tofacitinib, concluding that direct switching was appropriate in patients with RA. Similarly, another study in patients with RA found that switching from ADA to baricitinib, another JAK inhibitor, was associated with improvements in clinical disease control without increases in TEAEs, SAEs, or infections [15].

Consistent with these studies, the current analysis found that patients with PsA directly switching from blinded ADA to open-label tofacitinib achieved similar rates of ACR20/50 response, PASI75 response, PASDAS ≤ 3.2, and MDA response, and similar changes from baseline in FACIT-F, PGA-PsO, PASDAS, and Patient Assessment of Arthritis Pain (VAS) scores, compared with patients continuing tofacitinib 5 mg BID. In both groups, efficacy was maintained, and for some outcomes continued to improve, from the double-blind Phase 3 study to the open-label LTE study. While there were numerical differences between treatment groups for some outcomes, including ACR70, HAQ-DI, and VLDA responses, these did not consistently favor one treatment group, and differences between groups tended to decrease after treatment was switched in the LTE study. In the primary results of the Phase 3 study (OPAL Broaden), primary and secondary efficacy outcomes were similar with tofacitinib 5 mg BID and ADA, although the study was not designed to compare these two treatments directly [7]. Most patients who achieved ACR20/50/70 responses, PASI75 response, HAQ-DI response, PASDAS ≤ 3.2, or MDA/VLDA responses while receiving ADA also achieved these responses 3 months after switching to tofacitinib. Some patients who did not achieve responses for these outcomes after 12 months of treatment with ADA did achieve responses with tofacitinib after 3 months of treatment, supporting its value as a treatment option in patients who do not respond to TNFi.

Analyses of TEAEs, SAEs, and AEs of special interest demonstrated an acceptable safety profile in patients directly switching from ADA to tofacitinib 5 mg BID. Analyses of laboratory parameters did not find any clinically meaningful changes in patients directly switching from ADA to tofacitinib, or an increase in patients exceeding ULN thresholds, in the first 3 months after the treatment switch. In both treatment groups, rates of TEAEs were highest in the first 3 months of the Phase 3 study, as expected, and lower in the LTE study. In contrast, in the LTE study, SAE rates over the first 3 months and throughout the full 12 months were higher than in the Phase 3 study for both treatment groups but were only reported in small numbers of patients (≤ 7 per group). Similarly, AEs of special interest, including serious infections, herpes zoster, MACE, malignancies excluding NMSC, and NMSC occurred in few patients (≤ 2 per group), and there were no events of venous thromboembolism or deaths in either group. It should be noted that the study duration and sample size may have been insufficient to capture rare AEs that may have long latency, such as MACE and malignancies.

Limitations of this analysis include the fact that it was conducted post hoc, as the studies were not designed to compare ADA and tofacitinib, or to evaluate the impact of switching treatment. Furthermore, the sample size was small, and the timeframe analyzed was short. Data were analyzed as observed, with no imputation. Selection bias is possible as patients who entered the LTE study were those who completed the Phase 3 study or discontinued for reasons other than treatment-related AEs. Similarly, the patients who remained in the LTE study at months 3 and 6 may have been more likely to respond to tofacitinib. However, efficacy responses were generally similar across the time points examined, despite some reduction in the sample size over time.

## Conclusion

In conclusion, the efficacy and safety outcomes were generally similar in patients with PsA who directly switched from receiving ADA in a blinded Phase 3 study to receiving open-label tofacitinib 5 mg BID in an LTE study, compared with those who continued tofacitinib 5 mg BID in both studies. Patients in both groups maintained disease control over the first 3 (or 6) months of the LTE study and no increase in TEAEs was observed in the ADA→ tofacitinib 5 mg BID group after switching. These results suggest that patients with PsA can be directly switched from ADA to tofacitinib without any washout period.

## Electronic supplementary material

Below is the link to the electronic supplementary material.

Supplementary Material 1

## Data Availability

Upon request, and subject to review, Pfizer will provide the data that support the findings of this study. Subject to certain criteria, conditions, and exceptions, Pfizer may also provide access to the related individual de-identified participant data. See https://www.pfizer.com/science/clinical-trials/trial-data-and-results for more information.

## Abbreviations

ACR: American College of Rheumatology
ACR20/50/70: ≥ 20%/≥ 50%/≥ 70% improvements in American College of Rheumatology response criteria
ADA: Adalimumab
AE: Adverse event
ALT: Alanine aminotransferase
AST: Aspartate aminotransferase
bDMARD: Biological disease-modifying antirheumatic drug
BID: Twice daily
BMI: Body mass index
BSA: Body surface area
csDMARD: Conventional synthetic disease-modifying antirheumatic drug
DSS: Dactylitis severity score
FACIT-F: Functional Assessment of Chronic Illness Therapy-Fatigue
GT: Glutamyl transferase
HAQ-DI: Health Assessment Questionnaire-Disability Index
IR: Incidence rate
JAK: Janus kinase
LEI: Leeds Enthesitis Index
LS: Least squares
LTE: Long-term extension
MACE: Major adverse cardiovascular events
MDA: Minimal disease activity
MedDRA: Medical Dictionary for Regulatory Activities
NMSC: Non-melanoma skin cancer
P3: Phase 3
PASDAS: Psoriatic Arthritis Disease Activity Score
PASI: Psoriasis Area and Severity Index
PASI75: ≥75% Psoriasis Area and Severity Index improvement from baseline
PGA-PsO: Physician’s Global Assessment of Psoriasis
PsA: Psoriatic arthritis
PY: Patient-years
SAE: Serious adverse event
SAS: Safety analysis set
SD: Standard deviation
SE: Standard error
SOC: System Organ Class
TEAE: Treatment-emergent adverse event
TNFi: Tumor necrosis factor inhibitor
ULN: Upper limit of normal
VAS: Visual analog scale
VLDA: Very low disease activity
