# Supplementary material for: Efficacy and safety of tofacitinib in an open-label, long-term extension study in patients with psoriatic arthritis who received adalimumab or tofacitinib in a Phase 3 randomized controlled study: a post hoc analysis
Source: Arthritis Res Ther. 2024 Dec 19;26:218. doi: 10.1186/s13075-024-03442-2 (PMC11657006; doi:10.1186/s13075-024-03442-2)
Supplement: Supplementary file 1 — Supplementary Material 1 [file 13075_2024_3442_MOESM1_ESM.pdf]

Supplementary material

Supplementary Fig. 1 Additional efficacy outcomes in the Phase 3 and LTE studies

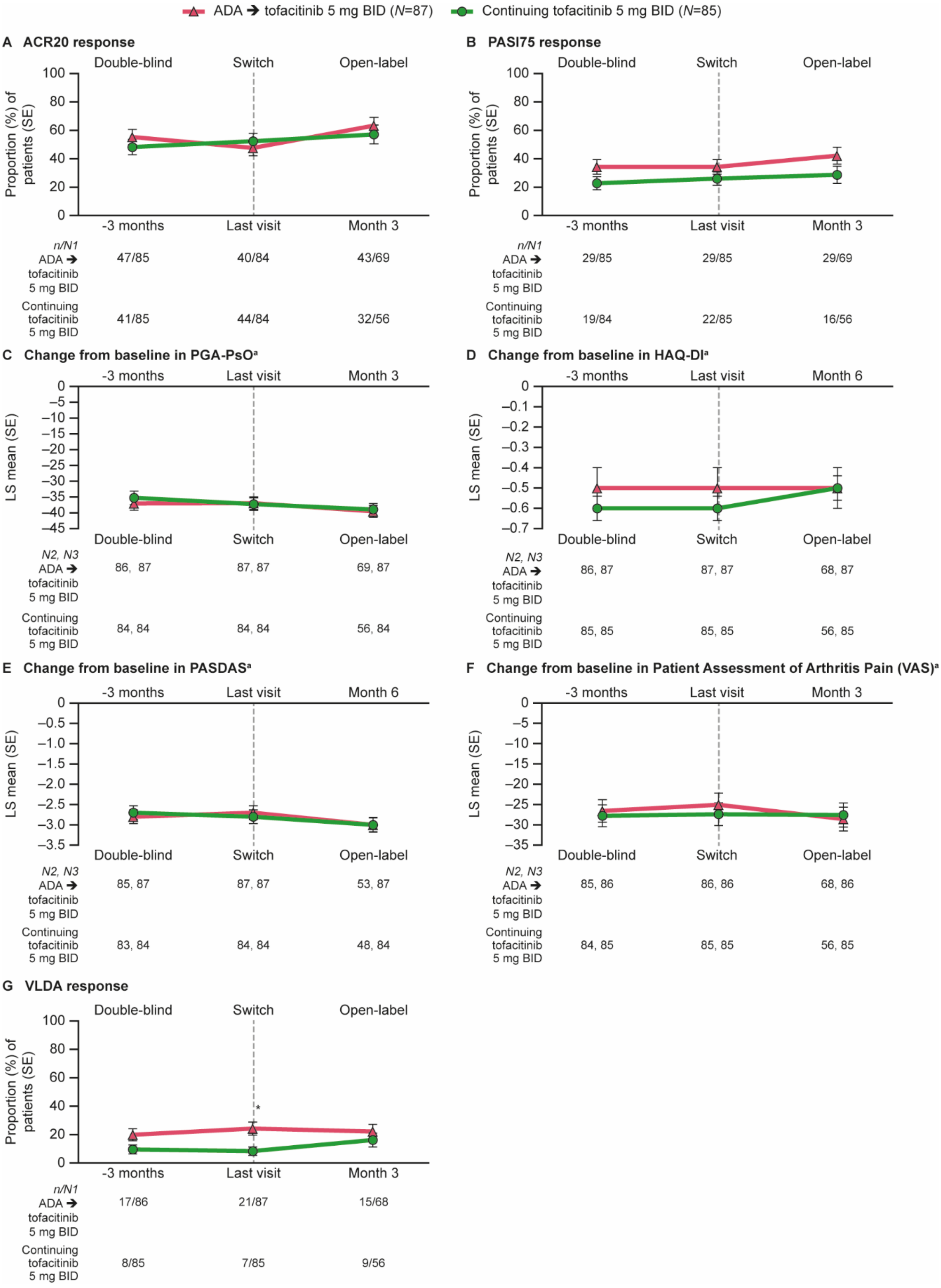

*$p<0.05$ for comparison between treatment groups.

-3 months: 3 months prior to the last visit in the Phase 3 study. Last visit: the last visit in the Phase 3 study. Month 3: month 3 in the LTE study. Month 6: month 6 in the LTE study. Baseline refers to the baseline visit of the Phase 3 study.

[a]Results are based on a repeated measures model with the fixed effects of treatment, visit, treatment by visit interaction, geographic location, and baseline value – an unstructured covariance matrix was used.

ACR50/70: ≥50%/≥70% improvement in American College of Rheumatology response criteria,
ADA: adalimumab, BID: twice daily, HAQ-DI: Health Assessment Questionnaire-Disability Index, LS: least squares, LTE: long-term extension, *N*: number of patients in the LTE SAS, *N1*: number of patients with non-missing response at visit, *N2*: number of patients with observations at visit, *N3*: number of patients included in the mixed model for repeated measures, *n*: number of responders, PASDAS: Psoriatic Arthritis Disease Activity Score, PGA-PsO: Physician's Global Assessment of Psoriasis, SAS: safety analysis set, SE: standard error, VAS: visual analogue scale, VLDA: very low disease activity.

Supplementary Fig. 2 Additional efficacy outcomes after switching from ADA→tofacitinib 5 mg BID according to Phase 3 ADA response

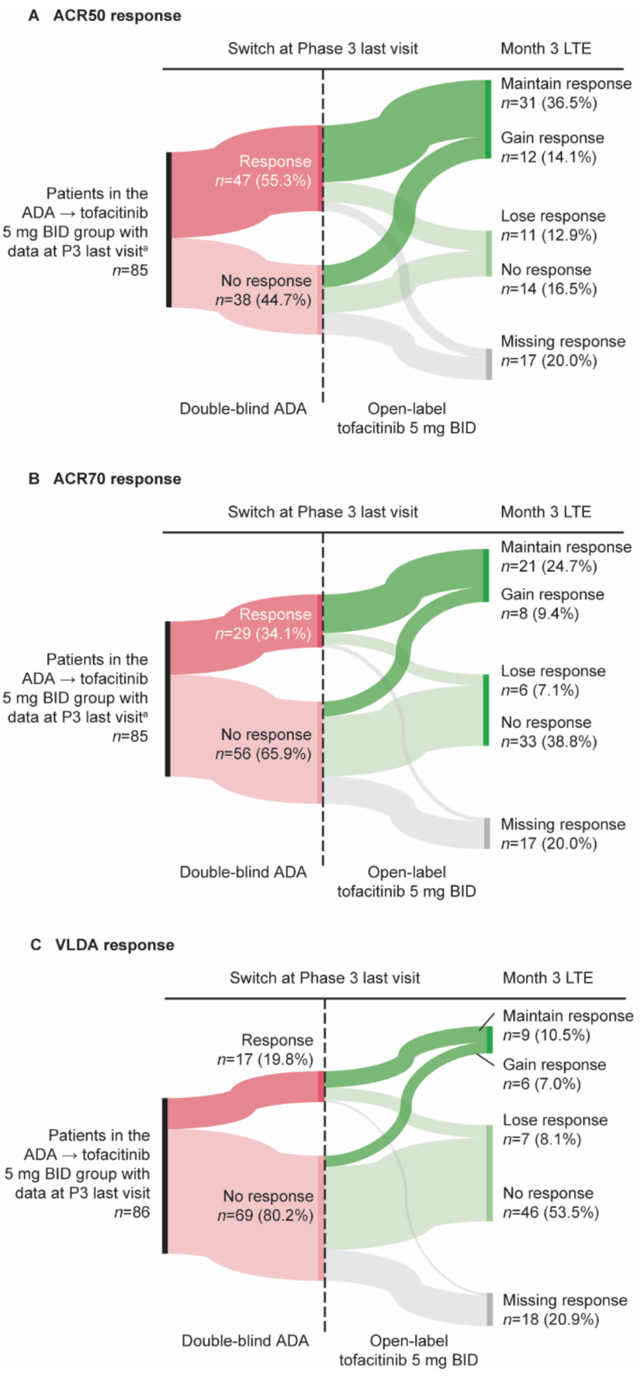

Efficacy outcomes for patients in the ADA→tofacitinib 5 mg BID group are shown following switch to tofacitinib in the LTE study according to ADA response in the Phase 3 study.

[a]Figure excludes one patient in the ADA→tofacitinib 5 mg BID group who had a missing response to ADA at the P3 last visit and no response to tofacitinib at the month 3 LTE.

ACR50/70: ≥50%/≥70% improvement in American College of Rheumatology response criteria,

ADA: adalimumab, BID: twice daily, LTE: long-term extension, *n*: number of patients in category, P3: Phase 3,

VLDA: very low disease activity.

Supplementary Fig. 3 Change from baseline in laboratory parameters in the Phase 3 and LTE studies

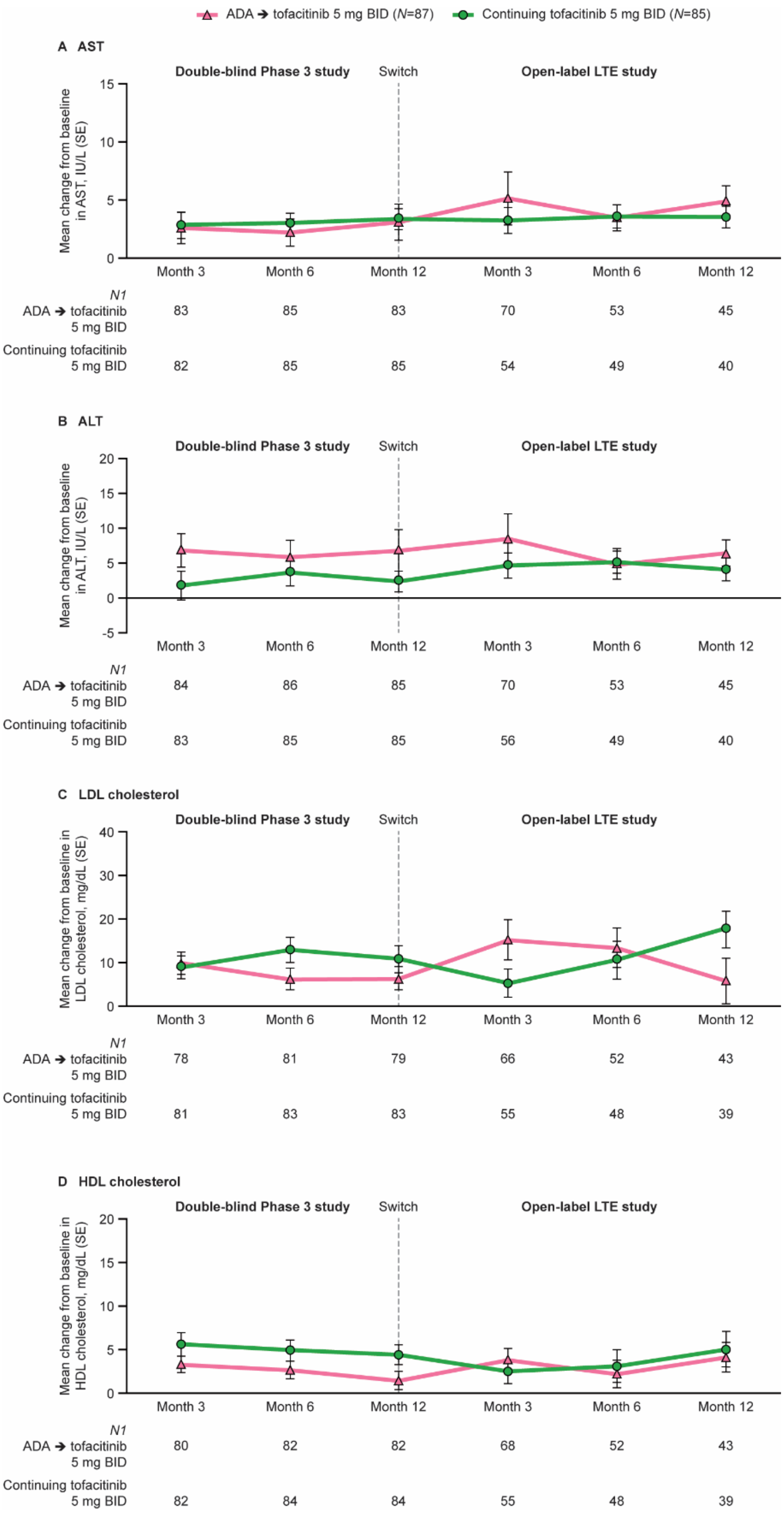

ADA → tofacitinib 5 mg BID (*N*=87)   Continuing tofacitinib 5 mg BID (*N*=85)

E Total cholesterol

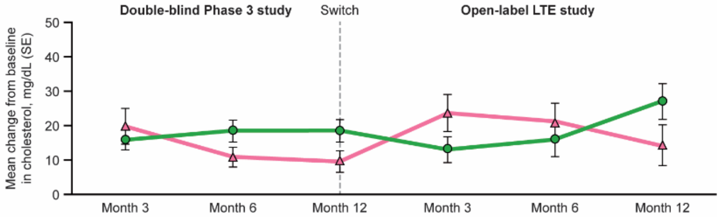

N1						
ADA → tofacitinib 5 mg BID	81	83	83	69	53	44
Continuing tofacitinib 5 mg BID	82	84	84	55	48	39

F Triglycerides

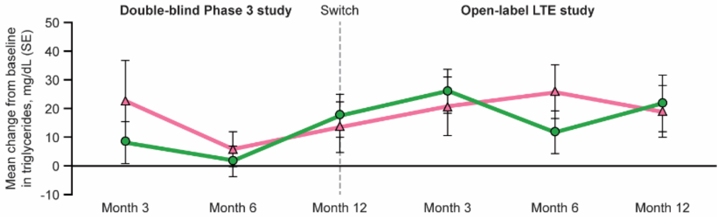

N1						
ADA → tofacitinib 5 mg BID	80	82	82	68	52	43
Continuing tofacitinib 5 mg BID	82	84	84	55	48	39

G Lymphocytes

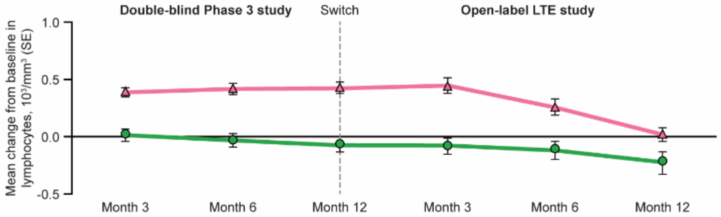

N1						
ADA → tofacitinib 5 mg BID	84	86	84	70	51	45
Continuing tofacitinib 5 mg BID	83	85	84	56	49	40

H Total neutrophils

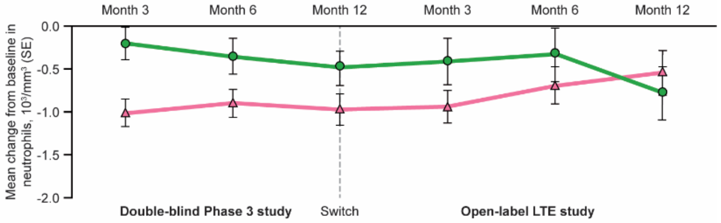

N1						
ADA → tofacitinib 5 mg BID	84	86	84	70	51	45
Continuing tofacitinib 5 mg BID	83	85	84	56	49	40

I Hemoglobin

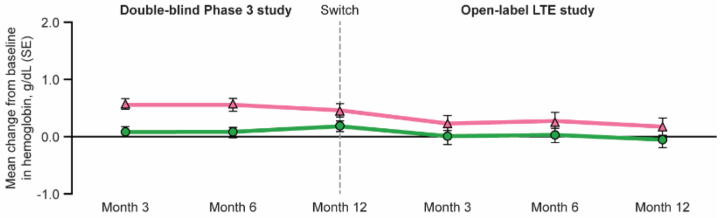

N1						
ADA → tofacitinib 5 mg BID	84	86	84	70	51	45
Continuing tofacitinib 5 mg BID	83	85	85	56	49	40

ADA: adalimumab, ALT: alanine aminotransferase, AST: aspartate aminotransferase, BID: twice daily, HDL: high-density lipoprotein, LDL: low-density lipoprotein, LTE: long-term extension, *N*: number of patients in the LTE SAS, *N1*: number of patients with observations at visit and baseline, SAS: safety analysis set, SE: standard error.

Supplementary Table 1 Incidence of laboratory parameters with thresholds in the Phase 3 and LTE studies

	**Phase 3 study**		**LTE study**	
	**ADA→ tofacitinib 5 mg BID (*N*=87)**	**Continuing tofacitinib 5 mg BID (*N*=85)**	**ADA→ tofacitinib 5 mg BID (*N*=80)**	**Continuing tofacitinib 5 mg BID (*N*=75)**
**Total bilirubin, *n* (%)**				
>1x ULN	7 (8.0)	4 (4.7)	4 (5.0)	3 (4.0)
≥2x ULN	0 (0.0)	0 (0.0)	0 (0.0)	2 (2.7)
≥3x ULN	0 (0.0)	0 (0.0)	0 (0.0)	1 (1.3)
≥5x ULN	0 (0.0)	0 (0.0)	0 (0.0)	1 (1.3)
≥10x ULN	0 (0.0)	0 (0.0)	0 (0.0)	0
**AST, *n* (%)**				
>1x ULN	26 (29.9)	26 (30.6)	18 (22.5)	10 (13.3)
≥2x ULN	10 (11.5)	4 (4.7)	2 (2.5)	7 (9.3)
≥3x ULN	3 (3.4)	2 (2.4)	2 (2.5)	2 (2.7)
≥5x ULN	1 (1.1)	0 (0.0)	0 (0.0)	2 (2.7)
≥10x ULN	0 (0.0)	0 (0.0)	0 (0.0)	1 (1.3)
**ALT, *n* (%)**				
>1x ULN	42 (48.3)	32 (37.6)	15 (18.8)	16 (21.3)
≥2x ULN	12 (13.8)	7 (8.2)	6 (7.5)	7 (9.3)
≥3x ULN	7 (8.0)	0 (0.0)	2 (2.5)	3 (4.0)
≥5x ULN	0 (0.0)	0 (0.0)	2 (2.5)	2 (2.7)
≥10x ULN	0 (0.0)	0 (0.0)	0 (0.0)	1 (1.3)
**Gamma GT, *n* (%)**				
>1x ULN	25 (28.7)	13 (15.3)	5 (6.3)	12 (16.0)
≥2x ULN	8 (9.2)	4 (4.7)	5 (6.3)	4 (5.3)
≥3x ULN	4 (4.6)	0	3 (3.8)	2 (2.7)
≥5x ULN	3 (3.4)	0	1 (1.3)	1 (1.3)
≥10x ULN	1 (1.1)	0	0	1 (1.3)

ADA: adalimumab, ALT: alanine aminotransferase, AST: aspartate aminotransferase, BID: twice daily, GT: glutamyl transferase, LTE: long-term extension, *N*: number of evaluable patients, *n*: number of patients with events, ULN: upper limit of normal.
